# Volatile organic compounds associated with *Plasmodium falciparum* infection in vitro

**DOI:** 10.1186/s13071-017-2157-x

**Published:** 2017-05-02

**Authors:** Ricardo Correa, Lorena M. Coronado, Anette C. Garrido, Armando A. Durant-Archibold, Carmenza Spadafora

**Affiliations:** 1 Center of Cellular and Molecular Biology of Diseases (CBCMe), Instituto de Investigaciones Científicas y Servicios de Alta Tecnología (INDICASAT AIP), City of Knowledge, Panama; 2 Department of Biotechnology, Acharya Nagarjuna University, Guntur, 522 510 AP India; 3 Molecular Medicine Research Unit, Center for Biodiversity and Drug Discovery, Instituto de Investigaciones Cientificas y Servicios de Alta Tecnologia (INDICASAT AIP), City of Knowledge, Panama

**Keywords:** Volatile organic compounds, HSPME, GC-MS, *Plasmodium falciparum*, Malaria, Vector

## Abstract

**Background:**

In order to identify new ways to prevent transmission of vector-borne diseases such as malaria, efforts have been made to understand how insects are attracted to humans. Vector-host interaction studies have shown that several volatile compounds play an important role in attracting mosquitoes to human targets. A headspace solid-phase micro-extraction/gas chromatography-mass spectrometry (HSPME GC-MS) analysis of the volatile organic composition of extracellular vesicles (EVs) and supernatants of ultracentrifugation (SNUs) was carried out in *Plasmodium falciparum-*infected cultures with high and low parasitemias.

**Results:**

A list of 18 volatile organic compounds (VOCs) was obtained from the EVs of both infected and uninfected RBCs with 1,2,3-Propanetriol, diacetate (diacetin) increased in the infected EVs, regardless of the parasitemia of the culture. The supernatant analysis, however, gave off 56 VOCs, with pentane 2,2,4-trimethyl being present in all the SNUs of uninfected erythrocytes but absent from the parasite-infected ones. Standing out in this study was hexanal, a reported insect attractant, which was the only VOC present in all samples from SNUs from infected erythrocytes and absent from uninfected ones, suggesting that it originates during parasite infection.

**Conclusions:**

The hexanal compound, reportedly a low-level component found in healthy human samples such as breath and plasma, had not been found in previous analyses of *P. falciparum-*infected patients or cultures. This compound has been reported as an *Anopheles gambiae* attractant in plants. While the compound could be produced during infection by the malaria parasite in human erythrocytes, the *A. gambiae* attraction could be used by the parasite as a strategy for transmission.

**Electronic supplementary material:**

The online version of this article (doi:10.1186/s13071-017-2157-x) contains supplementary material, which is available to authorized users.

## Background

*Plasmodium falciparum* is the protozoan parasite that causes the most severe variant of malaria cases worldwide [1]. The development of vector control strategies has been identified as an important pillar to decrease the malaria burden through transmission-reducing chemoprevention, correct insecticide applications and entomological surveillance [2]. Therefore, elucidating the mosquito’s role in malaria transmission is a key factor to understanding the deadliest worldwide vector-borne disease. In addition, the increase in drug-resistant parasites [3] and insecticide-resistant mosquitoes [4] is driving scientists and policy makers to develop alternative mechanisms to reduce the transmission of *P. falciparum.*

There have been many efforts to decode how mosquitoes are attracted to humans in order to look for new ways to stop transmission. Vector-host interaction studies have shown that several chemical compounds play an important role in attracting the *Anopheles* spp. to human targets. These chemical attractants include CO_2_ [5], octenol [6], indole [7], ammonia [8], lactic acid and aliphatic carboxylic acids [9, 10], which are contained in human breath and sweat. The role of skin-associated microflora VOCs has also been identified as a potential source of vector attraction [11, 12].

However, there is evidence that additional signal attractants must play a role during malaria infection. New findings identified an augmented attraction of mosquitoes to malaria-infected patients, suggesting a possible parasite manipulation on the physicochemical activity of the host [13–15] but this influence on humans by *P. falciparum* is not yet well understood.

Recent studies have been conducted to compare the volatile chemical burden during the asexual stage of *P. falciparum* in in vitro cultures. One study did not find any difference between *P. falciparum*-infected and non-infected RBCs [16]. However, a more recent report revealed the presence of several *P. falciparum-*associated terpenes. Notably, one of them, pinene, was found in very low quantities in infected red blood cells (iRBCs) [17]. Interestingly, pinene was previously reported as an *A. gambiae* attractant present in plants during sugar feeding [18]. Nevertheless, further analyses are needed to correlate that finding with the possibility of a host manipulation by the parasite to produce vector attraction signals during malaria infection.

The taking over of the host cell machinery by pathogens has been reported during several intracellular infections in bacteria, mammals and plants [19–22]. One such special control mechanism is the release of extracellular vesicles (EVs) to improve survival of the pathogen, as in macrophage infection by *Leishmania* parasites [23, 24]. The extracellular vesicles released from iRBCs during malaria infection have also been studied over the last few years, revealing that EVs are capable of transporting *Plasmodium* spp. molecules (RNA, DNA, protein and lipids) [25–29]. However, the VOC load in EVs has not been characterized in any malaria report thus far. Therefore, this study aimed to identify a possible differential content of EVs-VOCs in in vitro cultures of *P. falciparum* growing at two different parasitemias, and the probable relationship between the higher EV-VOC density and mosquito attraction.

## Methods

We cultured the *P. falciparum* HB3 strain using the conventional method of Trager & Jensen [30] with modifications described in Almanza et al. [31], that include the use of modified RPMI 1640 medium (Sigma-Aldrich, St. Louis, USA), 25 mM HEPES, 15 μM hipoxanthine, 50 mg/ml gentamicine sulfate, and 200 mM L-Glutamine, supplemented with 10% human serum, 2% sodium bicarbonate and a mix of gases (90% N_2_, 5% O_2_ and 5% CO_2_). Synchronization was performed in a temperature-cycling incubator (TCI) (Cooled Incubator, Sanyo, Model MIR-154) and by the addition of 0.3 M alanine (Sigma-Aldrich). Uninfected red blood cells (uRBCs) were cultured with the same hematocrit (2%) and media conditions.

Microvesicles were obtained from 25 ml of infected and uninfected RBCs from three volunteers. Supplementation media for each T75 culture bottle used the corresponding serum from each volunteer. Two replicas of uRBCs and of low (~4%) and high (15–30%) iRBC parasitemia were prepared from the blood of each volunteer. Parasitemia was evaluated by optical microscopy using Giemsa staining (GS500, Sigma-Aldrich). The procedure for isolation of microvesicles was based on a parasitic EV isolation report [32]. The iRBC and uRBC cultures were collected and centrifuged at 2000× *g* for 15 min. The 2000× *g* supernatants were then centrifuged at 15,000× *g* at 4 °C for 30 min to remove cell debris. Next, these supernatants were filtered through 0.2 μm low-binding protein filters (Acrodisc, Pall Life Science, Port Washington, USA) and the filtered-supernatants were ultracentrifuged at 110, 000× *g* 4 °C for 70 min to pellet small vesicles. The pellet was washed once by resuspending it in sterile double-filtered (0.2 μm) PBS 1X and further ultracentrifuged at 110,000× *g* for an additional 70 min. The pellet was resuspended in 100 μl of double-filtered PBS 1× for analysis, discarding the supernatant. At the same time, 20 ml of the supernatant formed in the first ultracentrifugation (SNU) were collected and concentrated to ~500 μl at 3000× *g* for 2 h using 3 kDa Vivaspin tubes.

The size-characterization of EVs by flow cytometry (CyFlow, Partec, Kent, UK) was performed using a similar procedure and parameters to those that have been used to measure microvesicles in plasma [33]. The EVs, which were characterized by measuring forward scatter (FSC) size and side scatter (SSC) granularity, were added immediately prior to analysis by flow cytometry. We established gates based on region size by calibrating the gain of polyethylene beads of different sizes (0.1, 0.5 and 2 μm) (Fluka Analytical, Sigma-Aldrich) before measuring the samples. We determined the background noise per second with 750 μl of double-filtered (0.2 μm) phosphate-buffered saline (PBS) solution. Data were acquired and analyzed using FloMax software. The final concentration of the samples was calculated using the software’s True Volumetric Absolute Counting system, based directly on the basic definition of concentration c = N/V, using an electrode-principle determination.

Independent cultures at high and low parasitemia were collected to perform western blot analysis in order to detect the presence of CD63 (System Biosciences, SBI, Exo AB kit-1, Palo Alto, USA), a conserved extracellular vesicle protein commonly used as an EV marker, and *Pf*MSP1 (Abcam, ab156840, Cambridge, UK) which is a merozoite membrane protein like the AMA1 used by Mantel et al. [29]. Also, Glycophorin A,B ( #G7650, Sigma-Aldrich), the main glycoprotein in the red blood cell membrane, was used. The antibodies were diluted as instructed by each manufacturer.

Samples were analyzed by HSPME-GC-MS. A 1.5 ml vial containing the sample was sealed and the SPME extraction was performed with a DVD/CAR/PDMS fiber (Supelco, Bellefonte, PA, USA). All HSPME extractions were done at 37 °C for 12 h. All samples were analyzed by gas chromatography-mass spectrometry analyses (GC-MS), using an Agilent 6890 N gas chromatographer connected to a 5975C triple-axis mass selective detector (Agilent Technologies, Palo Alto, CA, USA). Samples were injected in splitless mode, with the injector temperature set at 250 °C. The GC column used for the study was an HP-5MS, 30 m length, 0.25 mm i.d. and 0.25 μm phase thickness (Agilent Technologies, Palo Alto, CA, USA). Helium was used as carrier gas at 1.0 ml/min. The temperature gradient began with an initial temperature of 50 °C, held for 3 min, increased to 200 °C at 6 °C min^−1^, and finally upped to 280 °C at 10 °C min^−1^. Mass spectrometry detection was performed in the EI mode, with the ion source temperature, electron energy, and transfer line temperature set at 250 °C, 70 eV, and 280 °C, respectively. Identification of all compounds was done based on their fragmentation patterns using authentic standards when available, and the NIST 11 data base using mass spectral deconvolution and identification system (AMDIS). For further identification, the retention indices of each compound were compared with those reported in the literature.

## Results

The criteria used to choose the VOCs to which an abundance determination was applied was based on their presence in at least one of the technical replicates of all three volunteers. The comparison between their uRBCs and iRBCs levels was based on the detection of VOCs in the analysis of all samples [technical replicates of each parasitemia (2 high and 2 low) and 2 of uRBCs; all from 3 biological samples] from collected EVs, and their respective SNUs. In light of the limited amount of EVs collected from uRBCs, the two technical replicates of EVs from each volunteer were pooled before analyzing this population. This resulted in a total of 15 EV samples (3 uRBCs and 12 iRBCs) and 18 SNUs.

**Fig. 1:**
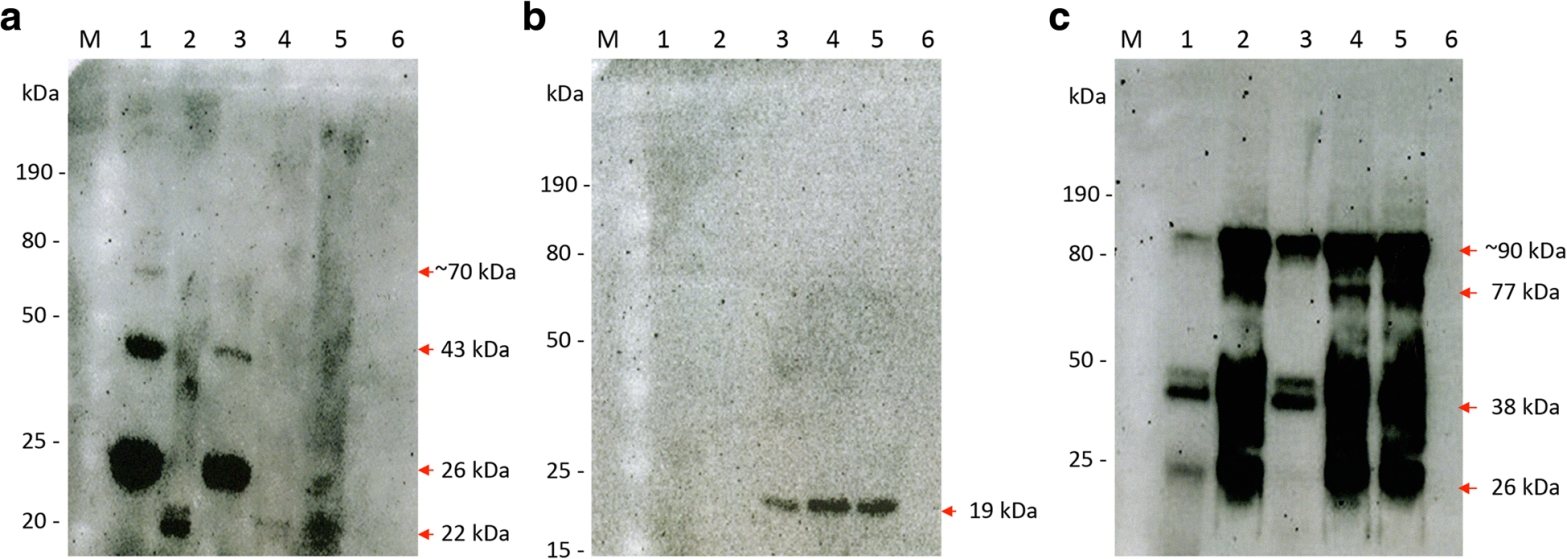
Extracellular vesicles (EVs) are recognized by common markers. Erythrocytes and EVs were lysed with RIPA buffer. Each lane was loaded with 20 μg of protein sample, as assessed by a Bradford assay, subjected to non-denatured (**a**) or SDS PAGE (**b**, **c**), transferred to polyvinyl membranes and probed with antibodies against CD63 (**a**), PfMSP1 (**b**) and Glycophorin A (**c**). CD63 specific primary antibodies were used at 1:1000 dilution and a secondary Goat anti-Rabbit IgG HRP conjugated antibody (System Bioscience) was used at 1:20,000 dilution. MSP1 was used at 1:50 dilution with a secondary Mouse IgG HRP-conjugated antibody (R&D System) at a 1:1000 dilution. To detect Glycophorin, the E3 clone was used following the manufacturer instructions. The gel lanes were loaded as follows: M: size marker; Lane 1: uninfected erythrocytes lysate; Lane 2: EVs from uninfected erythrocytes; Lane 3: infected erythrocytes lysate; Lane 4: EVs from infected erythrocytes at low parasitemia; Lane 5: EVs from infected erythrocytes at high parasitemia; Lane 6: Human serum. *Red* arrows mark the bands corresponding to the bands expected

The concentration of EVs varied from sample to sample (see Table 1 and Additional file 1: Table S1). Around 98% of the EVs had an average size of 0.1 μm when they were compared with standard microbeads. A reduced number (2%) of EVs fell in either the 0.5 μm or the 2 μm regions. In addition, the identity of the EVs after isolation was confirmed through detection of the human extracellular vesicle conserved marker CD63 and the parasite-specific membrane protein PfMSP1 by Western blot (Fig. 1). The typical bands below 30 kDa (one in duplet) [34], the 43 kDa [35] and a band above 50 kDa, as shown by the manufacturer, are all present in the blot. The MSP1 Western blot also reveals the expected 19 kDa band [36] (and in manufacturer’s site) only in lysates or EVs from infected cultures. In addition, Glycophorin A (GPA), a protein abundant in the membrane of red blood cells, was used to confirm the collected vesicles’ erythrocytic origin. A number of bands usually due to various glycosilation and dimerization states showed up in the blot [37], but most importantly, the EVs collected in this study were also rich in GPA and confirmed the source of the samples. The established protocol used to collect the EVs, their size determination by flow cytometry and the presence of GPA, PfMSP1 and CD63 in the population, altogether, confirm that our samples are extracellular vesicles from the parasite-infected erythrocytes.

**Table 1:** Concentration and size distribution of EV samples

	uRBCs	iRBCs Low Parasitemia	iRBCs High Parasitemia
Size	EV/ml (SD)	EV/ml (SD)	EV/μl (SD)
0.1 μm	74,331 (13,418.9)	77,970 (19,424.8)	200,232 (61,325.4)
0.5 μm	927 (812)	1094 (373.1)	1848 (1557.3)
2.0 μm	395 (295.5)	755 (362.2)	1326 (1053.7)

The identity of the VOCs found in this study was characterized after comparing the spectral fragmentation patterns with standards and subjecting the results to spectral deconvolution. Then, the retention indices, which are maintained across different types of columns, equipments and settings, were compared to those in the NIST 11 database (Table 2). In the analysis of EVs from iRBCs and uRBCs (Additional file 2: Table S2), one of the most abundant VOCs was 2-ethyl-1-Hexanol (Table 3), which is associated with plastic contamination [16]. Another major compound in our analysis was 1,3-bis(1,1-dimethylethyl)-Benzene, likely produced after gamma irradiation is applied to powder supplements in commercial RPMI [38]. There were no VOCs exclusive to EVs derived from iRBCs after analyzing all biological and technical replicas, although several molecules were present exclusively in one or more technical replicas of the iRBCs from a single volunteer (Additional file 2: Table S2).

**Table 2:** Experimental and theoretical retention indices of the VOCs

VOCs	Theoretical IR	Experimental IR
2,2,4-trimethyl-pentane	691	700
Hexanal	801	809
1-Octen-3-ol	979	987
Nonanal	1104	1111
2-ethyl-1-hexanol	1020	1028
Isoborneol	1160	1167
Menthol	1171	1179
α-Terpineol	1192	1200
Dodecane	1200	1208
1,2,3-propanetriol, diacetate	1230	1236
1,3-bis(1,1-dimethylethyl)-benzene	1249	1258
p-tert-butyl-phenol	1256	1262
Isobornyl acetate	1288	1296
Bornyl acetate	1290	1296
Tridecane	1300	1309
Tetradecane	1399	1407
β-Ionone	1489	1498
Butylated hydroxytoluene	1516	1523

**Table 3:** Abundance of VOCs of EVs (uRBCs and iRBCs). The total sum of all the peak areas of each compound in the chromatograms of all assays is listed. Hits are the number of replicates in which the compound was found

VOCs	Σ Area of uRBCs (hits out of 3 maximum replicates)	Σ Area of iRBCs (hits out of 12 maximum replicates)
2-ethyl-1-Hexanol	3,737,076 (3)	17,333,851 (12)
1,3-bis(1,1-dimethylethyl)- Benzene	3,737,076 (3)	13,753,288 (9)
1,2,3-Propanetriol, diacetate	1,509,468 (1)	12,786,280 (9)
Dodecane	967,212 (1)	5,347,592 (3)

Interestingly, 1,2,3-Propanetriol diacetate (Diacetin) was a commonly present compound on EVs from infected cultures, having at least 9 hits, reaching an average of 17.8% of the total parasitic EV VOCs according to the total area sum (Table 3)_._ Notably, while Diacetin was found in most samples from infected cultures, it also appeared in one technical replica of an uninfected volunteer. Other VOCs discovered only on the iRBCs were benzeneacetaldehyde, butanoic acid, butyl ester, ethylbenzene and o-xylene, however they only showed up once in our analysis, while several other compounds in the iRBCs, as shown in our databases, also showed up in most SNUs or EVs from uRBCs, as the alkenes undecane, tridecane, tetradecane and dodecane (Additional file 2: Table S2; Additional file 3: Table S3).

The analysis of SNUs was originally performed to distinguish VOCs in supernatants from those associated to the EVs. However, a higher number of VOCs was found in SNUs. We identified 56 different VOCs with variable frequencies (Additional file 3: Table S3). Only 17 compounds fulfilled the criteria for the analysis in this study (Table 4). The presence of the same contaminants found in the EV samples was also detected in SNUs. Of all the samples analyzed, hexanal appeared to be associated almost exclusively with SNUs from the iRBCs of all three volunteers, being absent in all replicas of uRBCs. Conversely, 2,2,4-trimethyl-pentane was found in all of the SNUs from the uRBCs but was completely absent from those of the iRBCs. Several terpenes such as isoborneol, borneol and menthol were often found in parasitized samples, possibly originating from the types of foods eaten by the volunteers prior to drawing their blood. These terpenes show a high variability among their relative areas, representing anywhere from 0.01 to 41.5% of them in any one sample. Notably, 1-octen-3-ol (a known mosquito attractant) was found to be increased in SNUs from iRBCs, although there was a high variability among samples.

**Table 4:** Abundance of VOCs from SNUs (uRBCs and iRBCs). The total sum of all the peak areas of each compound in the chromatograms of all assays is listed. Hits are the number of replicates in which the compound was found

VOCs	Σ Area of uRBCs (hits out of 6 maximum replicates)	Σ Area of iRBCs (hits out of 12 maximum replicates)
1,3-bis(1,1-dimethylethyl)-Benzene	175,199,553.0 (6/6)	280,310,131.0 (12/12)
Isoborneol	175,199,553.0 (6/6)	240,190,385.0 (11/12)
2-ethyl-1-Hexanol	175,199,553.0 (6/6)	280,310,131.0 (12/12)
Dodecane	175,199,553.0 (6/6)	280,310,131.0 (12/12)
Tetradecane	175,199,553.0 (6/6)	280,310,131.0 (12/12)
p-tert-butyl-Phenol	175199553.0 (6/6)	280,310,131.0 (12/12)
α-Terpineol	175,199,553.0 (6/6)	280,310,131.0 (12/12)
1-Octen-3-ol	140,850,914.0 (4/6)	280,310,131.0 (12/12)
Nonanal	175,199,553.0 (6/6)	212,397,351.0 (8/12)
Menthol	161,415,286.0 (5/6)	243,569,806.0 (11/12)
Isobornyl acetate	146,838,216.0 (4/6)	269,475,284.0 (11/12)
Hexanal	0/6	280,310,131.0 (12/12)
trans-β-Ionone	28,162,111.0 (1/6)	165,488,621.0 (7/12)
Tridecane	86,806,175.0 (1/6)	86,803,949.0 (3/12)
Bornyl acetate	28,361,337.0 (2/6)	10,834,847.0 (1/12)
2,2,4-trimethyl-pentane	175,199,553.0 (6/6)	(0/12)
Butylated hydroxytoluene	(0/6)	86,803,949.0 (3/12)

**Fig. 2:**
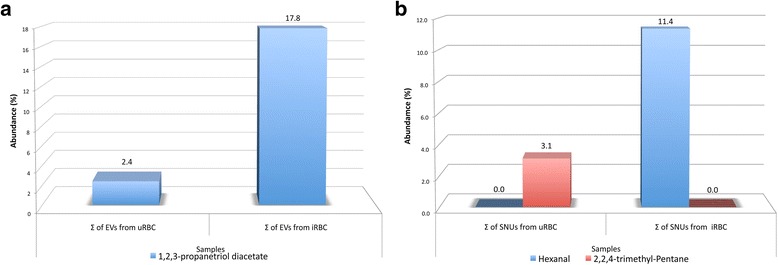
Difference in abundance of VOCs in *P. falciparum*-infected *vs* uninfected cultures. The peak areas of three volatile organic compounds, **a** 1,2,3-propanetriol diacetate (found in the EVs) from uRBC and iRBC and **b** 2,2,4-trimethyl-pentane and hexanal (found in SNUs) from uRBC and iRBC were summed up across all replicates and number of experiments. The total area thus found was compared between uninfected samples and infected ones

A graph of the three main findings of this study is presented in Fig. 2 where the difference in expression or production of each VOC is stressed.

## Discussion

The hypothesis of host manipulation by the parasite to induce mosquito-attractant VOCs through EVs was tested due to the capacity of the latter to transport key signal molecules. In this study, EVs isolated from *P. falciparum*-infected cultures, and the supernatants of those cultures at two different parasitemias, were analyzed for their VOC content. To validate the source of the compounds, immunoblots were used with common markers of extracellular vesicles [39]. The blots revealed that CD63 is enriched in all EVs, regardless of infection by the pathogen. The merozoite membrane protein MSP1, cleaved by serine proteases to give its 19 kDa size [36], is present only in extracellular vesicles from iRBCs, as expected, and is also present in the lysate of infected cultures. We have added a third marker to detect our extracellular vesicles: Glycophorin A. GPA is one of the most abundant proteins in the membrane of erythrocytic cells, and has numerous N- and O-glycosylation sites and also several polymorphic and quaternary structural presentations, making it migrate at different speeds on the polyacrylamide gels, depending on its state. We detected the presence of GPA in the cell lysates and in the EVs of all samples of red blood cell origin, demonstrating the usefulness of this marker as well for erythrocytic EVs.

As for the analysis of volatile material, a different methodology from those previously used to analyze standard or larger volumes of culture [16, 17] was used, which uncovered compounds not previously described as associated with *P. falciparum* or with its EVs. In addition, the use of human sera to culture the parasite offered the opportunity to perform VOC analysis in a more realistic environment in comparison with Albumax supplementation used in previous studies. In this scenario, the biological replicas of three human volunteers allowed us to understand conserved or non-conserved VOC production during infection by *P. falciparum.* The main obstacle faced in the study was the high concentration of possible contaminant compounds that could mask other less abundant VOCs. Most of these contaminants were present in both samples, suggesting an extrinsic origin derived from the culture media or plastic containers.

The high diversity of peak areas displayed by the replicas for each detected VOC implies a greater complexity than originally anticipated, which is independent of the level of parasitemia. The presence of diacetin in the vast majority of the EVs from iRBCs suggests a possible function in malaria infection. It is tempting to propose a role for this compound in the attraction of insects, given the recent report of diacetin being a phyto-attractant of oil bees [40], although more evidence is needed to verify this supposition. Additional compounds that were exclusive of the EVs from iRBCs did not have enough reproducibility to stand out given that they showed up in the iRBCs of only one out of the three volunteers.

The main difference in VOCs from uRBCs and iRBCs cultures was found in SNUs, even though the SNUs were initially intended to be used only as controls to differentiate the VOC-EV association. The terpenes found in our SNU samples were different from those reported to have mosquito attraction capacity, such as pinene [17]. Arguably, pinene and other terpenes not present in our samples - which contained only EVs or SNUs - could have their origin in the erythrocytes present in the sample when Kelly et al. [17] conducted their VOC search. This would explain why pinene, for example, has been found in blood from healthy volunteers [41]. Interestingly, the known solvent 2,2,4-trimethyl-pentane, present probably as a contaminant in the samples of uninfected RBCs, disappeared from the infected ones, as if the presence of the parasites had an influence on the decay of the compound.

As for the SNUs, hexanal was present in all of the samples of infected RBCs from both high and low parasitemia cultures. This compound was recently identified as a strong phyto-attractant of *A. gambiae* [18] and has been tested in mosquito baits [42]. Hexanal has been reported in several studies as a common marker in human breath and skin emanations, though these studies did not find it in the blood of healthy volunteers [41, 43–45]. Other technical approaches to the VOC content in humans, however, did find hexanal in normal plasma and breath, although at very low concentrations in comparison with the increased levels of hexanal connected to lung cancer patients [46–48], apparently linking its presence to cellular dysfunction or injury. Intriguingly, a recent study in humans infected with *P. falciparum* did not report hexanal in the breath of infected volunteers [49]. Although this VOC has been detected in in vitro cultures of *Plasmodium vinckei* [50], there have been as yet no reports on the generation of hexanal during *P. falciparum* infection. This compound is likely formed during membrane lipid peroxidation of cells as reported by Keller et al. [51], which would include RBCs in stress conditions [52] such as those encountered during parasitic infection. With its demonstrated attractiveness to the *Anopheles* mosquito, the production of hexanal could be advantageous to parasite transmission capabilities although studies are needed to test hexanal production in vivo in order to determine whether it plays a significant role in attracting mosquitoes to malaria-infected patients.

## Conclusions

An HSPME GC-MS analysis of extracellular vesicles and supernatants of *P. falciparum*-infected and uninfected RBCs was conducted. Close to 100 volatile organic compounds were detected which varied in their proportion and presence in the samples. Notably, diacetin, an insect attractant found in plants, was present in most of the extracellular vesicles of infected RBCs but found only once in the EVs of a healthy RBC sample, suggesting a possible role during malaria infection. Additionally, hexanal, not found in previous analyses of *P. falciparum-*infected patients, was present in our study in all supernatants coming from infected blood but absent in those from uninfected blood. It is noteworthy that hexanal has been described as an *A. gambiae* attractant in plants and, although it is possible that this compound is a by-product of the process of parasitic infection of the erythrocytes, its mosquito-attraction capabilities could be used by the parasite as a strategy to increase the likelihood of transmission. Although more studies are required in patients infected with *P. falciparum* to confirm these findings, this information could be significant in the development of strategies aimed at preventing transmission by offsetting the parasite’s vector-attracting capabilities.

## Additional files

Additional file 1: Table S1. Characterization by flow cytometry. Raw readings show the size of isolated extracellular vesicles (EVs from three volunteers (biological replicates). In addition, PBS 1X was also analyzed to eliminate the buffer background. Numbers were obtained after averaging the raw data. (XLSX 50 kb)

Additional file 2: Table S2. Detection of VOCs in extracellular vesicles (EVs). Raw data show the abundance area of each VOC in every sample from all volunteers. The total sample area is the sum of all samples. (XLSX 14 kb)

Additional file 3: Table S3. Detection of VOCs in SNUs. Raw data show the abundance area of each VOC in each sample from all volunteers. The total area sample was summed up for each sample. (XLSX 20 kb)

## Abbreviations

EV: Extracellular vesicle
HSPME GC-MS: Headspace solid-phase micro-extraction/gas chromatography-mass spectrometry
iRBC: Infected red blood cells
RBC: Red blood cells
SNU: Supernatant of ultracentrifugation
uRBC: Uninfected red blood cells
VOC: Volatile organic compound
